# Narrative writing as a strategy for nursing ethics education in Japan

**DOI:** 10.5116/ijme.5b39.d5d2

**Published:** 2018-07-23

**Authors:** Mari Tsuruwaka, Kiyomi Asahara

**Affiliations:** 1 Division of Bioethics, Graduate School of Nursing Sciences, St. Luke's International University, Japan; 2 Division of Public Health Nursing, Graduate School of Nursing Sciences, St. Luke's International University, Japan

**Keywords:** Narrative writing, nursing ethics education, graduate students, ethical issues, Japan

## Abstract

**Objectives:**

The purpose of this study was to explore the effectiveness of learning ethics of nursing practice using narrative writing.

**Methods:**

Study design was qualitative descriptive research. The participants were 90 graduate students who took nursing ethics classes, of whom 86 graduate students (4 males and 82 females) agreed to this study. The data gathered for analysis were their narratives described as feeling uncomfortable in clinical settings and their comment sheets after narrative group work in nursing ethics classes. We used qualitative content analysis to identify graduate students’ awareness gained through narrative writing and narrative group work.

**Results:**

As a feature of the scenes described by graduate students, there were often conflicts that patients’ autonomy were not respected, or that they were not able to engage in sincere engagement. The narrative writing was effective to make graduate students aware of the following two aspects: 1) habits and trends in one’s own thoughts and 2) organizational and administrative issues related to ethical issues.

**Conclusions:**

Learning ethics of nursing practice using narrative writing that focused on nurses’ sentiments helped reveal nurses’ thoughts as well as their attitudes and approaches to patients. These findings suggest that narrative writing in nursing ethics education could lead to ethical practice. Additionally, our results indicate that narrative writing in research may be helpful as a strategy to clarify ethical issues and the awareness of nurses in clinical settings.

## Introduction

The approaches most frequently used for considering clinical ethics have traditionally been based on ethical principles (respect for autonomy, nonmaleficence, beneficence, and justice)^1^ or have used frameworks that are represented by Jonsen’s four-box approach in Japan.^2^ These approaches identify complex ethical problems in clinical settings and introduce a solution for them. Among these methods, the narrative approach as a complementary way of considering medical ethics issues has received much attention.^3^^,^^4^

The narrative approach has been said to strengthen people’s ability to empathize and create interpersonal relationships by asking them to interpret and write a description of complex ethical situations.^5^ It requires people to assign meaning to a situation, to specific phenomena, or to a particular act. In doing so, the narrative approach is expected to promote deeper understanding and increase ethical sensitivity.^6^ In this approach, narratives are employed as a way to consider everyday healthcare ethics by focusing on the clinician’s emotions and not on his or her cognition.^7^ Narrative writing has also often become incorporated into the fields of nursing and medical education. Narrative writing is a subjective, literal expression of what we speak. One training program for medical residents was reported to have increased reflection and critical thinking through initiatives that asked trainees to compose their own narratives as well as those of their patients.^8^^,^^9^ In a particular study, first-year medical students conducting a geriatrics home visit were required to write an essay about the visit, which helped them to become aware of pertinent information in a specific personal context. ^10^ An educational program in which nurses wrote a narrative about their most memorable experiences as preceptors was reported to promote reflection on nursing practices and to cultivate growth.^11^ Carson’s educational program^12^ asked students to recall some point in their career when they felt conflict and to write a narrative about that experience; following that, in-class sessions, students formed groups and debated the problems described for approximately 30 minutes. When the students were requested to write about the same situation from the perspective of another person involved in the situation, they noticed that the conflict involved a combination of their personal choices and their professional duties.^12^ It has been indicated that narrative writing had not been actively incorporated into the curriculum because it would take a significant portion of the class time even though it would not be immediately applicable to clinical settings.^13^

The term *narrative* can carry a variety of meanings, such as literary works, fiction written by medical professionals, or descriptions of personal experiences. In the context of the ethical education of health care providers, the use of personal narratives as narrative writing has promoted ethical awareness among the people who write them^14^^,^^15^^,^^16^ guiding the writers to a deeper understanding of themselves or their patients and bringing their own personal experiences into the foreground.^12^ Because these narratives are personal scenarios, they enable their authors to recall details and vividly express the relevant facts.^17^ Further, reading these personal narratives can cause the reader to remember similar experiences.^18^ It is evident that protecting participants’ privacy requires attention when personal narratives are used in a classroom setting.^3^ Thus, when a narrative is used in a classroom, it is important to explain to the writer beforehand that it would be shared. A safe place where people can have discussions must be created.^12^

The scenes described through narrative writing depict a variety of relationships such as those between doctors and patients, doctors and the person writing the narrative, or doctors and society in general.^19^ Topics highlighted by medical residents have included the duty to respect the patient’s autonomy, the fair distribution of resources,^9^ and worrying about failure. Eight student nurses tend to write about uncomfortable experiences or clinical dilemmas.^20^ When clinicians were asked to write narratives that focused on negative emotions they had felt (e.g., anger, hate, or dejection), they often described ethical issues that were uncomfortable for them,^21^ as well as instances of conflict between the patient, other professionals, and families.^12^

It has been argued that the act of writing narratives is significant in three respects. First, the writers pursue the meaning of their experiences and reflect on their thoughts or behaviors.^11^^, ^^22^^, ^^23^^, ^^24^^, ^^25^^, ^^26^ Moreover, when others read and respond to the narrative, the discussion process promotes further reflection on the part of the writer.^16^ Second, writing narratives has increased ethical sensitivity. For example, in one study, writing a personal narrative about patient interactions in nursing practice caused the writers to notice ethical dilemmas in a clinical setting.^20^ It has also been reported that this process helped writers to recognize ethically important moments and increased their ethical mindfulness.^16^^,^^27^ Third, writing narratives were significant in that it appealed to writers’ emotions and not their cognition.^21^^,^^27^ Through the act of writing, the participants focused more strongly on their own emotions, questioning why they felt as they did and what they thought the patient felt.^20^

Nurses often experience unresolved feelings like “Was that really okay?” or “I thought that the situation wasn’t good, but nothing could be done about it.” These experiences are strongly intertwined with the lives of the people whom nurses serve. Conversations with nurses that we met in some classes or training sessions caused us to believe that such feelings were often suppressed within nurses themselves. The traditional problem-solving approaches normally used in clinical ethics are not, by themselves, sufficient to adequately describe the internal factors underlying the experiences of the people who harbor these uncomfortable feelings or the environments and situations that create these factors.

Narrative writing is being used more frequently in medical and nursing education, but few studies have empirically examined the application and effectiveness of narrative writing as an approach to learning clinical ethics.

Therefore, the purpose of this study was to explore the effectiveness of learning nursing ethics using narrative writing that was carried out in nursing ethics classes in a graduate school in Japan. Examination of awareness obtained from the graduate students’ experiences will contribute to help them discuss and analyze ethical issues in clinical settings.

### Initiatives in narrative work using narrative writing as a subject matter

The graduate school’s nursing ethics class is a half-term, two-credit, elective subject that meets for 135 minutes every week. We are in charge of nursing ethics classes. There are ten class sessions, and the content of the course consists of lectures that provide the foundation for considering ethics in nursing. Topics include ethical theory and principles, virtue ethics, an analytical method based on a systematic framework, practice in applying this framework, and the narrative approach.

The strategy of considering ethics in nursing that have applied the narrative approach usually contain two components: narrative writing and narrative group work. Narrative writing requires graduate students to recall uncomfortable scenes observed in clinical and practical training, to write their narratives from a subjective viewpoint, and to write one or two narratives of others who have something to do with the scene. This narrative writing was done individually, and it consisted of describing situations where people experienced uncomfortable feelings in a clinical setting. The graduate students wrote (1) an objective description of the scene (about 300 words), (2) their own personal narrative of this scene (about 300 words), and (3) a description of the presumed perspective of one or two other people who were involved in the scenes (about 200 words). Having the graduate students write a brief narrative from another person’s perspective was intended to help them think about how they perceived others, not to cause them to make inquiries as to what other people were thinking. The narrative work then consisted of in-class group discussions that used graduate students’ narrative writing as their subject matter. This narrative work took place in two of the ten class sessions as groups of about six graduate students each chose two scenes to examine. Because the narratives were to be shared with fellow class members, privacy considerations were emphasized, and graduate students were asked to change aspects of the story (e.g., the names of people involved) that were not essential to the story’s meaning.

The narrative group work had three objectives. The first was to enable graduate students to reflect on their actual nursing practices and to recognize and take an interest in the important moments in their everyday practice when ethical issues should be considered. The second objective was to have them examine from a variety of angles the factors and underlying contexts that create ethical issues. The third objective was to make graduate students more aware of their role, duties, and identity as healthcare professionals.

This narrative group work proceeded in the following way. Graduate students were asked to consider two tasks for about 60 minutes freely. As the first task, the group members were asked to thoroughly investigate points of concern or things that they found surprising and then to consider why they felt this way. Second, the graduate students were to consider the context within which each situation developed. After the group work of graduate students had completed these tasks, each group described what they had discussed with the entire class. Prior to the next class session, graduate students then filled out a comment sheet explaining what they had learned by using this method.

## Methods

### Study design and participants

We conducted qualitative descriptive research. The participants in this study were graduate students who took a nursing ethics class. The members of the nursing ethics class included some people with work experience as nursing professionals and some who had attained nursing qualifications but did not yet have any work experience. Ninety graduate students (5 males, 85 females) enrolled in the class over three years: 25 in 2013, 29 in 2014, and 36 in 2015. In this research, the narrative scenes described, and the comment sheets were used for analysis.

The graduate students attending the course each year received a written explanation of this study by hard copy and e-mail. The message explained the purpose of the study, its methods and ethical considerations, and the voluntary nature of participation. Those consenting to participate responded by sending a consent letter to the researchers’ address. The consent form included two separate indications about using the narrative scenes and comment sheets for research purposes. Personal information, such as people’s names, was deleted from the comment sheets and narrative scenes, which were numbered and anonymized before analysis, participants received written assurance that their personal information would not be specified in any way and that they would experience no disadvantage if they chose not to participate. We indicated that the anonymized data would be saved for at least five years after their publication in an academic journal and would then be destroyed. We further promised that if the data were published in an academic journal, participants’ anonymity would be preserved. This study was conducted with the approval of St. Luke’s International University’s Research Ethics Review Committee.

### Data collection

The data gathered for analysis were narratives described as feeling uncomfortable in clinical settings and comment sheets after narrative group work in nursing ethics classes by graduate students. Narrative scenes describe the feeling of discomfort, how I felt at that time, and so on. Also, the comment sheet was written about impressions of narrative writing and narrative group work itself. We explained the details of this study to the graduate students in written form and whether we could use the data as research. On that basis, we obtain the consent of the study participants.

### Data analysis

We used qualitative content analysis to explore graduate students’ awareness gained through narrative writing and narrative group work. The comment sheets were examined to assess the graduate students’ awareness about narrative group work. We repeatedly read the comments according to the context, which was written on the comment sheets after narrative group work, focusing on the graduate students' own narrative writing and their awareness about narrative group work. We extracted some codes that represented each context and named each code to express the main meaning of the content described. The codes were divided by the similarity of the meaning and redefined as a few decades of subcategories. After the subcategories were compared with each other and classified according to the similarity and correlation of the contents, several categories were defined with the degree of extraction increased. Newly added data was similarly compared with the existing categories and examined regarding how they were related with each other, with the degree of extraction increased to clarify the awareness of graduate students gained through narrative group work using narrative writing. We repeatedly discussed data collection and analysis to ensure the adequacy of coding. Additionally, we calculated the number of the narrative scenes as what kind of ethical issues occurred according to the Code of Ethics of Japan Nursing Association (2013).

## Results

Eighty-six narrative scenes and 130 comments described by 86 graduate students (4 males, 82 females) who gave consent

to the use of data as research materials were examined. The analysis was based on the following two points: 1) to clarify ethical issues that occurred in the uncomfortable scenes described, and 2) to explore the participants’ awareness gained from narrative group work using narrative writing.

### Characteristics of ethical issues of the narrative scenes written

In the 86 narrative scenes described, there were ethical issues as follows, according to the provisions of the Code of Ethics of the Japan Nursing Association.

The content in these scenes that caused the nurses to have uncomfortable feelings was as follows: not having an honest interaction (22%), not guaranteeing the right to know or right of self-determination (16%), not respecting the patient’s intentions (13%), not respecting the patient’s dignity as a human being (12%), denying the patient’s freedom to act (7%), not being able to protect the patient’s life (5%), not creating a relationship of mutual trust with the patient (5%), not understanding the patient’s indications (3%), not being able to fulfil the patient’s wishes (1%), not being able to provide benefits to the patient (1%), causing the patient to suffer pain (2%), not being able to interact with every patient equally (3%), other issues (10%).

These responses show that having honest interactions with patients and issues related to patient autonomy (e.g., right to know, right of self-determination, or patient intentions) were concerns in many of the cases that were described. There were cases in which the nurses struggled because they could not protect patients’ autonomy for various reasons and could not deal with the patients in a sincere manner. Concerns about honest interaction included a situation in which a terminal cancer patient began crying after the nurse had helped the patient to eat a meal, but the nurse had to leave quickly because of frequent nurse calls or morning handovers. In another typical example, a nurse described discomfort about having to wake up a patient in the intensive care unit at 1:00 a.m. and explain that the patient would have to move to another room because a new patient needed to be admitted to the ICU.

### Awareness gained from narrative work using narrative writing

An analysis of the insights gained from experience with narrative group work using narrative writing generated two core categories (theme): awareness of habits and trends in one’s own thoughts emerged and awareness of organizational and administrative issues identified (see Table 1).

#### Awareness of habits and trends in one’s own thoughts emerged

This core category extracted seven categories: being self-defensive; seeing things from an occupational viewpoint; thinking according to the convenience of medical professionals; having biased thoughts; denying negative emotions; wanting to build favorable relationships with the staff; being accustomed to the circumstances of the hospital and its organizational culture. Nineteen subcategories showing specific tendencies of thinking were extracted as well. These categories were articulated by showing some examples of subcategories below. The figures attached to the examples showed the number of each comment sheet.

#### Being self-defensive

This category means thoughts to protect oneself. Five specific subcategories were as follows: acting to evade denial and criticism; swallowing one’s words; trying to avoid troubles and litigation; giving up unavoidable matters; giving priority to safety.

##### Acting to evade denial and criticism:

My daily nursing practice is reflected in this nurse. When I saw the nurse denied, I felt I was denied, too. So, I noticed my emotional upset that made me try to defend the nurse to protect myself (Female, No. 037).

##### Swallowing one’s words:

I swallow my words very often. What I feel should be reviewed for patients and users cannot be seen when I swallow my words. I can do nothing when I find the atmosphere there doesn’t allow me to say something (Female, No. 078).

#### Seeing things from an occupational viewpoint

The occupational viewpoint indicates a view of nurses as a medical professional. Two specific subcategories were as follows: “having problem-solving thoughts”; “saving life first”.

##### Having problem-solving thoughts:

I am liable to think that problems must be solved, and I felt I was distressed by the thought (Female, No. 052).

#### Thinking according to the convenience of medical professionals

This category means thoughts not from the perspective of patients but from the viewpoint of medical providers. Three specific subcategories were as follows: being very busy; not thinking in a patient-centered manner; giving priority to efficiency.

##### Being very busy:

In clinical settings, I was distracted from uncomfortable scenes by being busy with work and care. I tried not to think about my sentiments by intention (Female, No. 009).

#### Having biased thoughts

This category implies that graduate students judged patients and families in a biased manner. Two specific subcategories were as follows: misconceiving; having preconceived ideas.

**Table 1:** Awareness gained from narrative writing

Core category	Category	Subcategory
Awareness of habits and trends in one’s own thoughts emerged	Being self-defensive	Acting to evade denial and criticism
		Swallowing one’s words
		Trying to avoid troubles and litigation
		Giving up unavoidable matters
		Giving priority to safety
	Seeing things from an occupational viewpoint	Having problem-solving thoughts
		Saving life first
	Thinking according to the convenience of medical professionals	Being very busy
		Not thinking in a patient-centered manner
		Giving priority to efficiency
	Having biased thoughts	Misconceiving
		Having preconceived ideas
	Denying negative emotions	Feeling remorseful
		Feeling guilty
	Wanting to build favorable relationships with the staff	Not disturbing the relationship
		Refraining from expressing oneself
		Being fully aware of what other people think of
	Being accustomed to the circumstances of the hospital and its organizational culture	Not having an uncomfortable feeling
		There is a difference of awareness between staff and patients living daily life
Awareness of organizational and administrative issues emerged	Power relationship is a contributory factor	There is a power relationship within a team
		There are power relationships among nurses
	The system and culture within organizations is a contributory factor	There aren’t enough human resources
		Management system is insufficient
		The nature of organizations is undesirable
		The policies of organizations are undesirable
		The culture of organizations is undesirable
	Social conditions are contributory factors	The systems of hospitals in accepting patients from other medical institutions are undesirable
		The systems of law and society are undesirable

#### Misconceiving:

Actually, I was looking at my patients from behind a filter. This attitude made me misconceive what they needed (Female, No. 043).

### Denying negative emotions

This category means that it is not desirable that nurses have negative emotions toward patients. Two specific subcategories were as follows: feeling remorseful; feeling guilty.

#### Feeling guilty:

Negative emotions make me feel guilty quite often, and it is difficult to express that feeling (Female, No. 025).

### Wanting to build favorable relationships with the staff

This category implies that building good relations with the staff is a top priority, even though there are other matters to consider. Three specific subcategories were as follows: not disturbing the relationship; refraining from expressing oneself; being fully aware of what other people think of.

#### Not disturbing the relationship:

I do not want to disturb the relationship, I do not want to cause troubles, so I will refrain from telling it to the doctor. I

won’t share it with others (Female, No.129).

#### Refraining from expressing oneself:

There were some occasions in which I didn’t express myself to keep a good relationship among staff. I strongly felt we were linked with each other in our society (Female, No. 041).

### Being accustomed to the circumstances of the hospital and its organizational culture

This category indicates thoughts arising from environments of the hospital and its organizational culture, very familiar and common to the staff. Two specific subcategories were as follows: not having an uncomfortable feeling; there is a difference of awareness between staff and patients living daily life.

#### Not having an uncomfortable feeling:

Because I have been working at the same place for a long time, I am accustomed to the culture of the place and don’t question what I felt earlier was awkward (Female, No. 080).

#### There is a difference of awareness between staff and patients living daily life:

I am convinced that the status quo cannot be helped. Even a little experience of practical training and nursing education can distract me from how general people feel. My perception that the environment of hospitals and medical services are peculiar has become stronger (Female, No. 074).

### Awareness of organizational and administrative issues emerged

This core category extracted three categories and nine subcategories showing specific organizational and administrative matters. Three categories were as follows: power relationship is a contributory factor; the system and culture within organizations is a contributory factor; social conditions are contributory factors. We described categories by showing some examples of subcategories below.

### Power relationship is a contributory factor

This category indicated that power relationship exists behind ethical issues. Two specific subcategories were as follows: there is a power relationship within a team; there are power relationships among nurses.

#### There are power relationships among nurses:

Even though the relations of some nurses looked good and friendly, power relationship within them seemed to exist. I became aware of the background of the organization (Female, No. 030).

### The system and culture within organizations is a contributory factor

This category referred to the relationship between the system of hospital organizations and the culture of organizations, and ethical issues. Five specific subcategories were as follows: there aren’t enough human resources; management system is insufficient; the nature of organizations is undesirable; the policies of organizations are undesirable; the culture of organizations is undesirable.

#### The nature of organizations is undesirable:

I was unfavorably impressed by the closed organization. Everything is settled only within each ward (Female, No 088).

#### The culture of organizations is undesirable:

I found that the system and culture of the organization had a big influence on my thoughts (Female, No. 045).

### Social conditions are contributory factors

This category referred to the fact that ethical issues were linked with social factors. Two specific subcategories were as follows: the systems of hospitals in accepting patients from other medical institutions are undesirable; the systems of law and society are undesirable.

#### Undesirable systems of hospitals in accepting patients from other medical institutions:

I felt that social factors such as undesirable systems of hospitals in accepting patients from other medical institutions were very influential. Due to various limitations of social factors, actions and responses taken by organizations are decided (Female, No. 003).

These results indicated that narrative group work using narrative writing revealed participants’ (graduate students) attitudes and approaches in situations where ethical issues occurred. The graduate students who performed narrative work began to recognize how they interacted with patients and how they tended to act, as well as ethical issues of unprotected rights and privacy of patients. Meanwhile, it has also become apparent that the issues faced by the organizations to which they belong and various other social factors are related to their attitudes and approaches. Nurses frequently became defensive due to their organization’s policies, their relationships with fellow nurses, or their power relationship with doctors. In some cases, participants found out that they refrained from expressing themselves because they wanted to create or maintain favorable relationships.

## Discussion

This strategy (narrative writing and narrative group work) revealed the nurses' own thinking patterns and perceptions, as well as the background of ethical issues, especially organizational and administrative challenges. Awareness of their thoughts and perceptions leads to encouraging reflection. It has also been reported in some previous studies that narrative writing promotes reflection.^11^^,^^22^^,^^23^^,^^24^^,^^25^^,^^26^ Through the act of writing, the writers can access their emotions and not just their cognition^21^^,^^27^ leading them to continue reflecting on and questioning why they felt as they did.^20^ Since the assignment asked the nurses to write about how they felt in settings when negative emotions were aroused in them - and not simply to compose an objective version of the facts - it helped them to uncover and confront their own emotions. Thus, it enabled participants to consider deeply not only various types of ethical issues but also how they participated in them. For example, some nurses experienced uncomfortable feelings when a doctor gave an inadequate explanation to a patient. Writing about the experience subjectively caused the nurses to reflect

on their own behavior and ask themselves questions, such as why they did not take action as nursing professionals to protect the patient’s right to know in such a situation. The nurses then were able to focus on the context and factors that prevented them from taking action. It has been noted that narrative writing previously, by enabling people to stop and consider how they felt and acted in a situation, helps to reveal their core values and priorities.^28^

Writing subjectively, in the first person, about one’s emotions enables people to observe inconsistencies between their thinking and their behavior, or to become aware of tendencies in their own thought. People have the opportunity to reflect on whether their discovery is something that they indeed hadn’t seen previously or something that they had intentionally tried not to notice. In modern ethics education, epistemological approaches have emphasized ethical thinking and decision making but have not given much consideration to human emotions. In some cases, in fact, such approaches have excluded consideration of the role of hidden emotions in ethical decision making.^21^ In their line of work, nurses are generally expected to maintain objective viewpoints and assessments and to solve problems based on objective information. In contrast, the narrative approach’s focus on subjectivity promotes thinking about limitations of or issues with a person’s natural way of thinking. In doing so, it causes the nurses to realize that certain things cannot be seen from a purely objective viewpoint.

The method that we have applied here is not limiting by nature; instead, it thoroughly investigates what problems were present in a particular scene that someone was concerned about. Rather than forcing participants into a box, this approach cultivates free thinking. It also seems to be connected with refining ethical sensitivity. Approaches based on ethical principles allow people to learn an ethical framework, but such approaches often cause people to justify their behavior rather than deepening their ethical thinking.^29^ Employing solely principle-based ethical thinking does not allow practitioners to cultivate human relationships that can open up a wide variety of possibilities.^30^ In the process of doing narrative group work, the writers attempt to understand the situations of other people by taking new perspectives and considering a variety of experiences, and in doing so, they are constantly pushed to ask “why” questions. This process enables them to see perspectives that they had not understood up to that point or things that they had intentionally put a lid on. Additionally, the participants in narrative group work come to recognize that other nurses have had similar experiences, and they understand that their own experiences are not unique but rather frequently occur about relating to a variety of factors. The participants are then able to focus on these factors. It has been pointed out that ethical issues that occur in clinical settings are related to environmental and administrative problems of organizations^31.32 ^and that they deepen moral distress of nurses.^33.34^ Verkerk et al. proposed three reflection steps to encourage moral awareness in ethics education for medical providers. In that framework, “social norm” (the code of ethics for professionals, laws, and moral beliefs) and “consequences” (social structure, practices of people’s lives, and culture) were accounted important factors.^35^ In other words, thinking about ethical issues occurring in clinical settings required looking at organizational environments, culture, and administrative matters. The narrative group work using narrative writing presented in this paper made us aware of organizational and administrative problems as well as social factors, which caused uncomfortable feelings in everyday nursing practice. The strategy has the power to help these challenges.

The narrative scenes described by participants included some cases of complicated medical decisions that had a serious impact on patients’ lives. However, the nurses were more frequently concerned about having honest interactions with their patients or how to respect patients as people in their everyday nursing practices - for example, in their daily communication with patients or when providing help with maintenance of hygiene. It thus seems that the narrative method helps nurses to perceive the presence of ethical problems in their ordinary, daily nursing practices. Benner noted that ordinary ethical and clinical approaches are determined by what constitutes best practice in normal, everyday cases and not by the extremely difficult cases.^30^

Several related issues should be investigated further through individual interviews and additional, more thorough studies. Questions deserving further consideration include how the use of narrative writing in ethics impels people to reflect on their own practices up to that point, how this strategy changes their cognition, and how their new insights are applied to their subsequent practice. As mentioned in previous studies, a key to effective use of this technique is to create a safe space^12^ for discussions of personal narratives, especially given the possibility that a participant who authored a narrative expressing dissatisfaction with his or her prior behavior could feel self-hatred or become depressed. Employing this technique requires sufficient sensitivity to the possible emergence of unexpected emotions.

The limits of this study were the following three points: 1) It is an attempt done at one university in Japan, and it is difficult to generalize, 2) It is dependent on the curriculum of the preliminary learning and nursing ethics, 3) The possibility that Japanese culture and health care system may have influenced the results of the study.

## Conclusions

This study examined the effectiveness of using narrative writing as a means of enhancing instructions in nursing ethics through the educational practices of a graduate school in Japan. This approach led graduate students to fully recognize their own tendencies of thoughts and their attitudes and approaches toward patients. It also enabled nurses to perceive more clearly organizational and administrative issues that were associated with ethical challenges. These findings suggest that using narrative writing in nursing ethics education stimulates thinking about everyday ethics in health care, which means that it could lead to ethical practice. Additionally, our results indicate that narrative writing in research may be helpful as a strategy to clarify ethical issues and the tendencies of thinking of nurses in clinical settings.

### Acknowledgments

We would like to thank all the participants who contributed to this study. The present study was supported by Grants-in-Aid for Young Scientist (B) for 2012-2014 (24790514) from the Japan Society for the Promotion of Science.

### Conflict of Interest

The authors declare that they have no conflict of interest.
